# Supplementary material for: Phenolic Constituents from Wendlandia tinctoria var. grandis (Roxb.) DC. Stem Deciphering Pharmacological Potentials against Oxidation, Hyperglycemia, and Diarrhea: Phyto-Pharmacological and Computational Approaches
Source: Molecules. 2022 Sep 13;27(18):5957. doi: 10.3390/molecules27185957 (PMC9505740; doi:10.3390/molecules27185957)
Supplement: Supplementary file 1 [file molecules-27-05957-s001.zip › molecules-1872691-supplementary.pdf]

Supplementary file

Phenolic Constituents from *Wendlandia tinctoria* var. *grandis* (Roxb.) DC. Stem Deciphering Pharmacological Potentials Against Oxidation, Hyperglycemia and Diarrhea: Phyto-Pharmacological and Computational Approaches

Mamtaz Farzana [1], Md. Jamal Hossain [2,*], Ahmed M. El-Shehawi [3], Md. Al Amin Sikder [1], Mohammad Sharifur Rahman [1], Muhammad Abdullah Al-Mansur [4], Sarah Albogami [3], Mona M. Elseehy [5], Arpita Roy [6], M. Aftab Uddin [7], and Mohammad A. Rashid [1,*]

[1] Department of Pharmaceutical Chemistry, Faculty of Pharmacy, University of Dhaka, Dhaka 1000, Bangladesh
[2] Department of Pharmacy, State University of Bangladesh, Dhanmondi, Dhaka 1205, Bangladesh
[3] Department of Biotechnology, College of Science, Taif University, P.O. Box 11099, Taif 21944, Saudi Arabia
[4] Bangladesh Council of Scientific and Industrial Research (BCSIR), Dr. Qudrat-I-Khuda Road, Dhanmondi, Dhaka-1205, Bangladesh
[5] Department of Genetics, Faculty of Agriculture, University of Alexandria, Alexandria 21545, Egypt
[6] Department of Biotechnology, School of Engineering & Technology, Sharda University, Greater Noida 201310, India
[7] Department of Genetic Engineering and Biotechnology, Faculty of Biological Sciences, University of Dhaka, Dhaka-1000, Bangladesh
* Correspondence: jamal.du.p48@gmail.com or jamalhossain@sub.edu.bd (M.J.H.); arpharm64@du.ac.bd or r.pchem@yahoo.com (M.A.R.); Tel.: +880-1517-814-866 (M.J.H.); +880-1711-947-741 (M.A.R.)

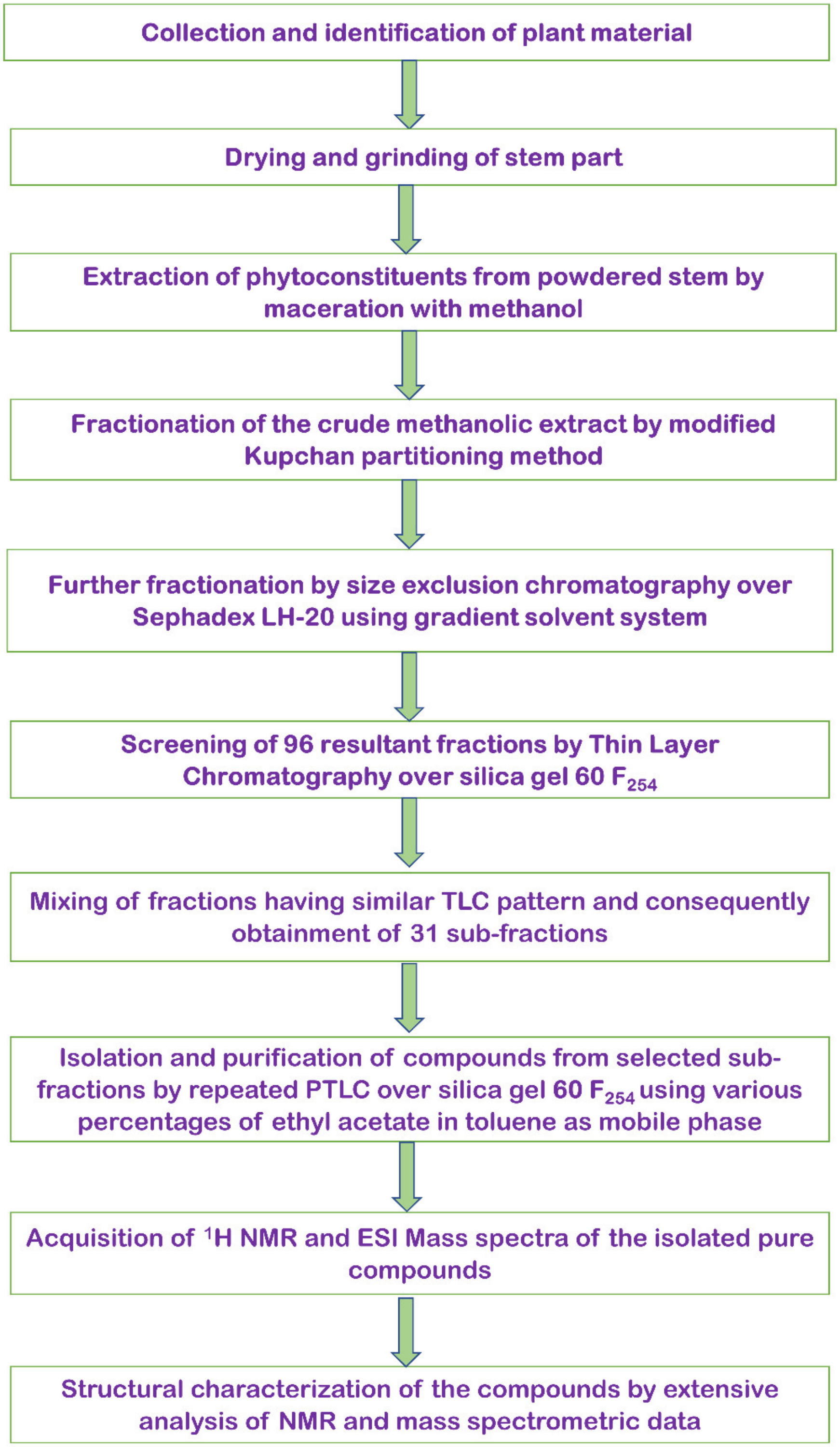

Figure S1: The schematic diagram of whole phytochemical isolations.

Table S1. Gradient solvent system used in size exclusion chromatographic technique.

Solvent System	Fraction Numbers*	Total Volume Collected (mL)
Hexane : DCM : Methanol (2 : 5 : 1)	1-17	51
10% Methanol in DCM (10 : 90 = 1 : 9)	18-35	54
20% Methanol in DCM (20 : 80 = 1 : 4)	36-53	54
50% Methanol in DCM (50 : 50 = 1 : 1)	54-71	54
80% Methanol in DCM (80 : 20 = 4 : 1)	72-89	54
100% Methanol	90-96	21

*Fractions were collected in test tubes. 3 mL of eluate had been collected in each test tube.

Table S2. Mixing of fractions based on similar TLC pattern.

**Fraction Numbers**	**Sub-fraction**
1-5	F-1
6-9	F-2
10-13	F-3
14-17	F-4
18-20	F-5
21-23	F-6
24-26	F-7
27-29	F-8
30-31	F-9
32-33	F-10
34-35	F-11
36-39	F-12
40-43	F-13
44-47	F-14
48-50	F-15
51-53	F-16
54-56	F-17
57-60	F-18
61-63	F-19
64-65	F-20
66-68	F-21
69-71	F-22
72-74	F-23
75-77	F-24
78-79	F-25
80-81	F-26
82-83	F-27
84-85	F-28
86-87	F-29
88-89	F-30
90-96	F-31

Table S3. Isolation of compounds by PTLC technique from different sub-fractions.

Sub-fraction	Solvent System of Preparative TLC Technique	Isolated Compounds	Amount of Pure Compounds (mg)
F-23	50 % Ethyl Acetate in Toluene	Compound **6** (Ferulic acid)	4.0
F-24	55% Ethyl Acetate in Toluene	Compound **5** (Glabridin)	4.3
F-25	55% Ethyl Acetate in Toluene	Compound **1** (Liquiritigenin)	4.5
F-26	50 % Ethyl Acetate in Toluene	Compound **2** (Naringenin)	4.1
F-27	55% Ethyl Acetate in Toluene	Compound **3** (Apigenin)	4.2
F-28	50 % Ethyl Acetate in Toluene	Compound **8** (4-Hydroxybenzaldehyde)	3.8
F-29	55% Ethyl Acetate in Toluene	Compound **7** (4-Hydroxybenzoic acid)	3.9
F-30	60 % Ethyl Acetate in Toluene	Compound **4** (Kaempferol)	4.6

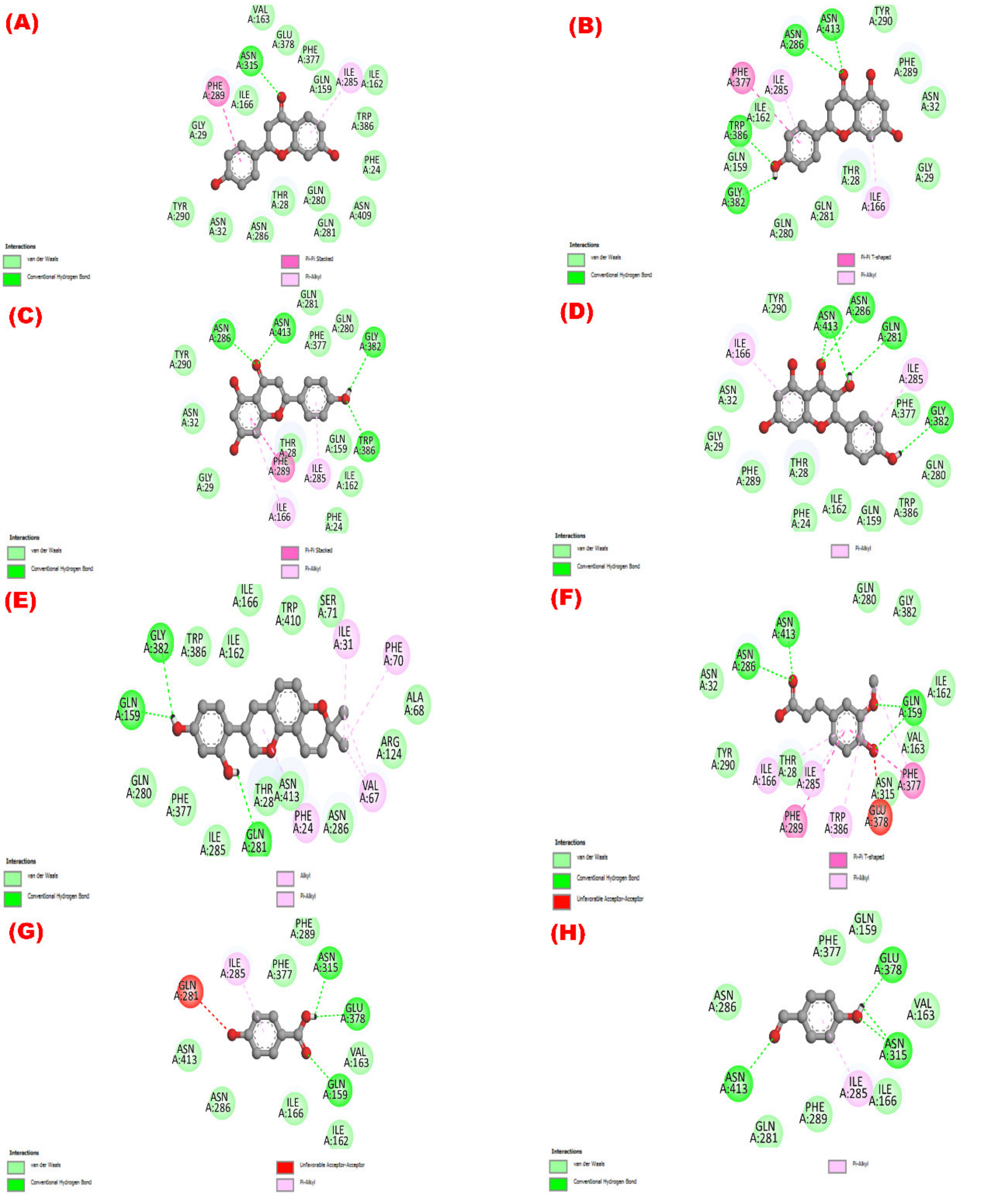

Figure S2. The isolated phytochemicals' potential as hypoglycemic agents showing their 2D molecular interactions with the glucose transporter 3 (GLUT 3) (PDB ID: 4ZWB). (The 2D visual images of the molecular docking of the molecules (**1** to **8**) liquiritigenin, naringenin, apigenin, kaempferol, glabridin, ferulic acid, 4-hydroxybenzoic acid, and 4-hydroxybenzaldehyde are shown in A, B, C, D, E, F, G, and H, respectively).

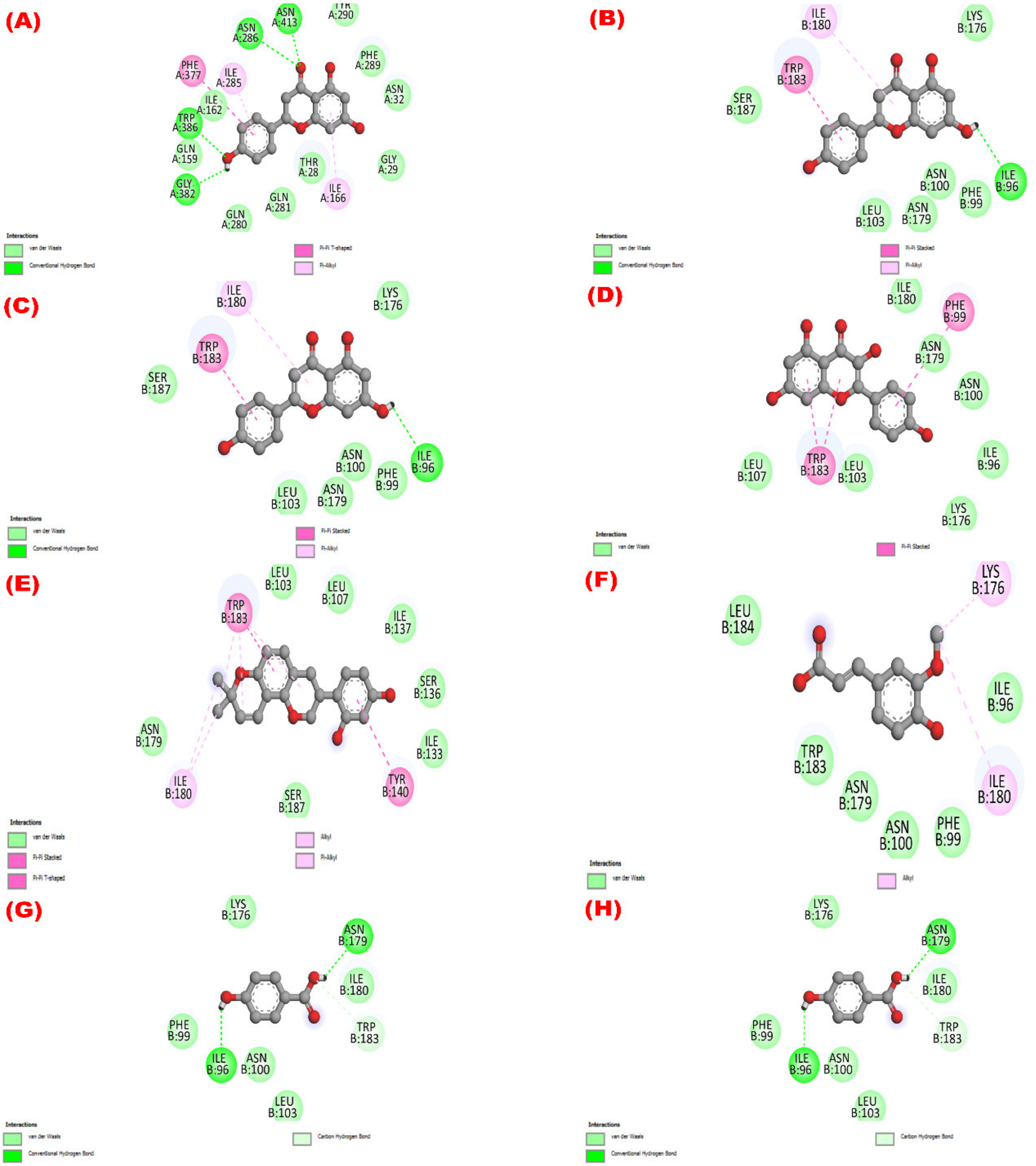

Figure S3. The isolated phytochemicals' potential as antidiarrheal agents showing their 2D molecular interactions with the kappa opioid receptor (PDB ID: 6VI4). (The 2D visual images of the molecular docking of the molecules (**1** to **8**) liquiritigenin, naringenin, apigenin, kaempferol, glabridin, ferulic acid, 4-hydroxybenzoic acid, and 4-hydroxybenzaldehyde are shown in A, B, C, D, E, F, G, and H, respectively).

Table S4: Interactions of amino acids of the glutathione reductase enzyme (PDB ID: 3GRS) with the isolated compounds (**1** to **8**) from *Wendlandia tinctoria* (Roxb.) DC. and the standard drug Butylated Hydroxy toluene (BHT) during molecular docking.

Compound	Bond (AA and Ligand)	Bond length	Bond type	Bond nature
Liquiritigenin (**1**)	A:SER51:HN - N:UNK1:O	2.75893	Hydrogen Bond	Conventional Hydrogen Bond
	A:GLU50:CA - N:UNK1:O	3.53249	Hydrogen Bond	Carbon Hydrogen Bond
	A:GLU50:OE2 - N:UNK1	4.4349	Electrostatic	Pi-Anion
	A:THR156:CG2 - N:UNK1	3.44276	Hydrophobic	Pi-Sigma
	N:UNK1 - A:LEU298	5.44018	Hydrophobic	Pi-Alkyl
Naringenin (**2**)	A:SER51:HN - N:UNK1:O	2.53482	Hydrogen Bond	Conventional Hydrogen Bond
	A:GLU50:OE2 - N:UNK1	4.48616	Electrostatic	Pi-Anion
	A:THR156:CG2 - N:UNK1	3.49392	Hydrophobic	Pi-Sigma
	A:HIS129 - N:UNK1	4.7889	Hydrophobic	Pi-Pi Stacked
	N:UNK1 - A:LEU298	5.43351	Hydrophobic	Pi-Alkyl
Apigenin (**3**)	N:UNK1:H - A:GLU50:OE1	2.13242	Hydrogen Bond	Conventional Hydrogen Bond
	A:ASP331:OD1 - N:UNK1	4.20119	Electrostatic	Pi-Anion
	N:UNK1 - A:ALA342	5.01057	Hydrophobic	Pi-Alkyl
Kaempferol (**4**)	A:SER51:HN - N:UNK1:O	3.08093	Hydrogen Bond	Conventional Hydrogen Bond
	N:UNK1:H - A:THR57:OG1	2.1136	Hydrogen Bond	Conventional Hydrogen Bond
	N:UNK1:H - N:UNK1:O	2.2186	Hydrogen Bond	Conventional Hydrogen Bond
	N:UNK1:H - A:ASN294:OD1	2.91263	Hydrogen Bond	Conventional Hydrogen Bond
	A:GLU50:OE2 - N:UNK1	4.33452	Electrostatic	Pi-Anion
	A:THR156:CG2 - N:UNK1	3.49098	Hydrophobic	Pi-Sigma
	A:HIS129 - N:UNK1	4.71522	Hydrophobic	Pi-Pi Stacked
	N:UNK1 - A:LEU298	4.93945	Hydrophobic	Pi-Alkyl
Glabridin (**5**)	A:ASN294:HD21 - N:UNK1:O	2.6422	Hydrogen Bond	Conventional Hydrogen Bond
	N:UNK1 - A:MET159	5.40639	Hydrophobic	Alkyl
	N:UNK1:C - A:VAL61	4.0771	Hydrophobic	Alkyl
	N:UNK1:C - A:VAL61	5.21401	Hydrophobic	Alkyl
	N:UNK1:C - A:PRO160	5.09846	Hydrophobic	Alkyl
	A:HIS52 - N:UNK1	5.34463	Hydrophobic	Pi-Alkyl
Ferulic acid (**6**)	A:GLY31:HN - N:UNK1:O	2.29547	Hydrogen Bond	Conventional Hydrogen Bond
	A:THR339:HN - N:UNK1:O	2.75951	Hydrogen Bond	Conventional Hydrogen Bond
	N:UNK1:H - A:ASP331:OD2	2.33705	Hydrogen Bond	Conventional Hydrogen Bond
	N:UNK1:H - A:ALA155:O	2.18953	Hydrogen Bond	Conventional Hydrogen Bond
	N:UNK1:C - A:VAL329:O	3.44338	Hydrogen Bond	Carbon Hydrogen Bond
	A:GLY29:C,O;SER30:N - N:UNK1	4.73456	Hydrophobic	Amide-Pi Stacked
	A:ALA155 - N:UNK1:C	3.33905	Hydrophobic	Alkyl
	A:ALA342 - N:UNK1:C	4.18634	Hydrophobic	Alkyl
	N:UNK1:C - A:VAL329	5.35381	Hydrophobic	Alkyl
	N:UNK1 - A:ALA342	5.27267	Hydrophobic	Pi-Alkyl
4-Hydroxybenzoic acid	A:SER51:HN - N:UNK1:O	3.08093	Hydrogen Bond	Conventional Hydrogen Bond

(**7**)	N:UNK1:H - A:THR57:OG1	2.1136	Hydrogen Bond	Conventional Hydrogen Bond
	N:UNK1:H - N:UNK1:O	2.2186	Hydrogen Bond	Conventional Hydrogen Bond
	N:UNK1:H - A:ASN294:OD1	2.91263	Hydrogen Bond	Conventional Hydrogen Bond
	A:GLU50:OE2 - N:UNK1	4.33452	Electrostatic	Pi-Anion
	A:THR156:CG2 - N:UNK1	3.49098	Hydrophobic	Pi-Sigma
	A:HIS129 - N:UNK1	4.71522	Hydrophobic	Pi-Pi Stacked
4-Hydroxybenzaldehyde (**8**)	N:UNK1 - A:LEU298	4.93945	Hydrophobic	Pi-Alkyl
	A:SER30:HN - N:UNK1:O	2.2803	Hydrogen Bond	Conventional Hydrogen Bond
	A:THR57:HN - N:UNK1:O	2.62161	Hydrogen Bond	Conventional Hydrogen Bond
	A:CYS58:HN - N:UNK1:O	2.19969	Hydrogen Bond	Conventional Hydrogen Bond
	A:GLY157:HN - N:UNK1:O	2.9589	Hydrogen Bond	Conventional Hydrogen Bond
	N:UNK1:H - A:VAL329:O	2.59624	Hydrogen Bond	Conventional Hydrogen Bond
	N:UNK1 - A:ALA342	4.76081	Hydrophobic	Pi-Alkyl
Butylated Hydroxy Toluene (BHT)	N:UNK1:H - A:GLY158:O	2.63579	Hydrogen Bond	Conventional Hydrogen Bond
	N:UNK1:C - A:LYS53	4.38415	Hydrophobic	Alkyl
	N:UNK1:C - A:VAL61	4.80028	Hydrophobic	Alkyl

Table S5: Interactions of amino acids of the urase oxidase enzyme (PDB ID: 1R4U) with the isolated compounds (**1** to **8**) from *Wendlandia tinctoria* (Roxb.) DC. and the standard drug Butylated Hydroxy toluene (BHT) during molecular docking.

Compound	Bond (AA and Ligand)	Bond length	Bond type	Bond nature
Liquiritigenin (**1**)	A:PRO76:CD - N:UNK1:O	3.64553	Hydrogen Bond	Carbon Hydrogen Bond
	A:MET32:SD - N:UNK1	5.5761	Other	Pi-Sulfur
	A:CYS103:SG - N:UNK1	3.64542	Other	Pi-Sulfur
	A:TYR30 - N:UNK1	5.02944	Hydrophobic	Pi-Pi T-shaped
	N:UNK1 - A:PRO76	5.24592	Hydrophobic	Pi-Alkyl
	N:UNK1 - A:ARG105	4.43966	Hydrophobic	Pi-Alkyl
Naringenin (**2**)	N:UNK1:H - A:VAL73:O	2.67752	Hydrogen Bond	Conventional Hydrogen Bond
	A:PRO76:CD - N:UNK1:O	3.69628	Hydrogen Bond	Carbon Hydrogen Bond
	A:PRO76:CD - N:UNK1:O	3.21635	Hydrogen Bond	Carbon Hydrogen Bond
	A:MET32:SD - N:UNK1	5.42942	Other	Pi-Sulfur
	A:CYS103:SG - N:UNK1	3.64118	Other	Pi-Sulfur
	A:TYR30 - N:UNK1	5.03267	Hydrophobic	Pi-Pi T-shaped
	N:UNK1 - A:PRO76	5.41381	Hydrophobic	Pi-Alkyl
	N:UNK1 - A:ARG105	4.42633	Hydrophobic	Pi-Alkyl
Apigenin (**3**)	N:UNK1:H - A:VAL73:O	2.38223	Hydrogen Bond	Conventional Hydrogen Bond
	A:PRO76:CD - N:UNK1:O	3.23331	Hydrogen Bond	Carbon Hydrogen Bond
	A:MET32:SD - N:UNK1	5.44523	Other	Pi-Sulfur
	A:CYS103:SG - N:UNK1	3.66988	Other	Pi-Sulfur
	A:TYR30 - N:UNK1	5.68215	Hydrophobic	Pi-Pi T-shaped
	A:TYR30 - N:UNK1	5.00672	Hydrophobic	Pi-Pi T-shaped
	N:UNK1 - A:PRO76	5.43513	Hydrophobic	Pi-Alkyl
	N:UNK1 - A:CYS103	4.40463	Hydrophobic	Pi-Alkyl
	N:UNK1 - A:ARG105	4.29356	Hydrophobic	Pi-Alkyl
Kaempferol (**4**)	A:TRP106:HN - N:UNK1:O	2.09023	Hydrogen Bond	Conventional Hydrogen Bond
	N:UNK1:H - A:VAL73:O	1.68644	Hydrogen Bond	Conventional Hydrogen Bond
	N:UNK1:H - A:VAL29:O	2.20195	Hydrogen Bond	Conventional Hydrogen Bond
	N:UNK1:H - A:THR107:OG1	2.36105	Hydrogen Bond	Conventional Hydrogen Bond
	A:MET32:SD - N:UNK1	5.20113	Other	Pi-Sulfur
	A:CYS103:SG - N:UNK1	3.60193	Other	Pi-Sulfur
	A:TYR30 - N:UNK1	5.0092	Hydrophobic	Pi-Pi T-shaped
	N:UNK1 - A:ARG105	4.06825	Hydrophobic	Pi-Alkyl
	N:UNK1 - A:PRO76	5.27194	Hydrophobic	Pi-Alkyl
Glabridin (**5**)	N:UNK1:H - A:GLU31:O	2.46697	Hydrogen Bond	Conventional Hydrogen Bond
	A:MET32:SD - N:UNK1	5.55821	Other	Pi-Sulfur
	A:CYS103:SG - N:UNK1	3.80112	Other	Pi-Sulfur
	A:TYR30 - N:UNK1	4.84733	Hydrophobic	Pi-Pi T-shaped
	A:TRP208 - N:UNK1	5.21519	Hydrophobic	Pi-Alkyl
	N:UNK1 - A:PRO76	5.27332	Hydrophobic	Pi-Alkyl

Ferulic acid (**6**)	A:ARG128:HH11 - N:UNK1:O	2.65297	Hydrogen Bond	Conventional Hydrogen Bond
	A:ARG128:HH12 - N:UNK1:O	2.7048	Hydrogen Bond	Conventional Hydrogen Bond
	N:UNK1:H - A:ASP205:OD2	2.63582	Hydrogen Bond	Conventional Hydrogen Bond
	N:UNK1:C - A:VAL29:O	3.63376	Hydrogen Bond	Carbon Hydrogen Bond
	A:MET32:SD - N:UNK1	5.55891	Other	Pi-Sulfur
	A:CYS103:SG - N:UNK1	3.73779	Other	Pi-Sulfur
	A:TYR30 - N:UNK1	4.9124	Hydrophobic	Pi-Pi T-shaped
	A:TYR30 - N:UNK1:C	5.23047	Hydrophobic	Pi-Alkyl
	N:UNK1 - A:PRO76	5.17868	Hydrophobic	Pi-Alkyl
4-Hydroxybenzoic acid (**7**)	A:PRO76:CD - N:UNK1:O	3.48004	Hydrogen Bond	Carbon Hydrogen Bond
	A:MET32:SD - N:UNK1	5.80393	Other	Pi-Sulfur
	A:CYS103:SG - N:UNK1	3.80232	Other	Pi-Sulfur
	A:TYR30 - N:UNK1	4.92498	Hydrophobic	Pi-Pi T-shaped
	N:UNK1 - A:PRO76	5.24399	Hydrophobic	Pi-Alkyl
4-Hydroxybenzaldehyde (**8**)	A:PRO76:CD - N:UNK1:O	3.48004	Hydrogen Bond	Carbon Hydrogen Bond
	A:MET32:SD - N:UNK1	5.80393	Other	Pi-Sulfur
	A:CYS103:SG - N:UNK1	3.80232	Other	Pi-Sulfur
	A:TYR30 - N:UNK1	4.92498	Hydrophobic	Pi-Pi T-shaped
	N:UNK1 - A:PRO76	5.24399	Hydrophobic	Pi-Alkyl
Butylated Hydroxy Toluene (BHT)	N:UNK1:C - A:TRP208	3.90117	Hydrophobic	Pi-Sigma
	N:UNK1:C - A:CYS103	4.42683	Hydrophobic	Alkyl
	N:UNK1:C - A:ARG105	4.80501	Hydrophobic	Alkyl
	N:UNK1:C - A:ARG128	4.8105	Hydrophobic	Alkyl
	A:TYR30 - N:UNK1:C	5.16445	Hydrophobic	Pi-Alkyl

Table S6: Interactions of amino acids of the glucose transporter 3 (GLUT 3) (PDB ID: 4ZWB) with the isolated compounds (**1** to **8**) from *Wendlandia tinctoria* (Roxb.) DC. and the standard drug glibenclamide during molecular docking.

Compound	Bond (AA and Ligand)	Bond length	Bond type	Bond nature
Liquiritigenin (**1**)	A:ASN315:HD21 - N:UNK1:O	2.42169	Hydrogen Bond	Conventional Hydrogen Bond
	A:PHE289 - N:UNK1	5.00603	Hydrophobic	Pi-Pi Stacked
	N:UNK1 - A:ILE285	2.69255	Hydrogen Bond	Conventional Hydrogen Bond
Naringenin (**2**)	A:ASN286:HD22 - N:UNK1:O	2.51237	Hydrogen Bond	Conventional Hydrogen Bond
	A:TRP386:HE1 - N:UNK1:O	2.13081	Hydrogen Bond	Conventional Hydrogen Bond
	A:ASN413:HD21 - N:UNK1:O	2.92491	Hydrogen Bond	Conventional Hydrogen Bond
	N:UNK1:H - A:GLY382:O	5.21554	Hydrophobic	Pi-Pi T-shaped
	A:PHE377 - N:UNK1	5.25385	Hydrophobic	Pi-Alkyl
	N:UNK1 - A:ILE166	4.96032	Hydrophobic	Pi-Alkyl
	N:UNK1 - A:ILE285	2.74792	Hydrogen Bond	Conventional Hydrogen Bond
Apigenin (**3**)	A:ASN286:HD22 - N:UNK1:O	2.74792	Hydrogen Bond	Conventional Hydrogen Bond
	A:TRP386:HE1 - N:UNK1:O	2.50964	Hydrogen Bond	Conventional Hydrogen Bond
	A:ASN413:HD21 - N:UNK1:O	2.09824	Hydrogen Bond	Conventional Hydrogen Bond
	N:UNK1:H - A:GLY382:O	2.83774	Hydrogen Bond	Conventional Hydrogen Bond
	A:PHE289 - N:UNK1	5.08793	Hydrophobic	Pi-Pi Stacked
	N:UNK1 - A:ILE166	5.27196	Hydrophobic	Pi-Alkyl
	N:UNK1 - A:ILE285	5.21415	Hydrophobic	Pi-Alkyl
Kaempferol (**4**)	A:ASN286:HD22 - N:UNK1:O	2.81097	Hydrogen Bond	Conventional Hydrogen Bond
	A:ASN413:HD21 - N:UNK1:O	2.0868	Hydrogen Bond	Conventional Hydrogen Bond
	A:ASN413:HD21 - N:UNK1:O	2.24774	Hydrogen Bond	Conventional Hydrogen Bond
	N:UNK1:H - A:GLY382:O	3.06051	Hydrogen Bond	Conventional Hydrogen Bond
	N:UNK1:H - A:GLN281:OE1	2.78735	Hydrogen Bond	Conventional Hydrogen Bond
	N:UNK1 - A:ILE166	5.17702	Hydrophobic	Pi-Alkyl
	N:UNK1 - A:ILE285	5.32515	Hydrophobic	Pi-Alkyl
Glabridin (**5**)	N:UNK1:H - A:GLN281:OE1	2.79923	Hydrogen Bond	Conventional Hydrogen Bond
	N:UNK1:H - A:GLN159:OE1	1.99103	Hydrogen Bond	Conventional Hydrogen Bond
	N:UNK1:H - A:GLY382:O	2.91833	Hydrogen Bond	Conventional Hydrogen Bond
	N:UNK1:C - A:VAL67	4.67268	Hydrophobic	Alkyl
	N:UNK1:C - A:ILE31	5.38653	Hydrophobic	Alkyl
	N:UNK1:C - A:VAL67	4.45202	Hydrophobic	Alkyl
	A:PHE24 - N:UNK1	5.13569	Hydrophobic	Pi-Alkyl
	A:PHE70 - N:UNK1:C	5.06367	Hydrophobic	Pi-Alkyl
Ferulic acid (**6**)	A:GLN159:HE21 - N:UNK1:O	2.15033	Hydrogen Bond	Conventional Hydrogen Bond
	A:GLN159:HE21 - N:UNK1:O	2.74877	Hydrogen Bond	Conventional Hydrogen Bond
	A:ASN286:HD22 - N:UNK1:O	2.62438	Hydrogen Bond	Conventional Hydrogen Bond
	A:ASN413:HD21 - N:UNK1:O	1.87569	Hydrogen Bond	Conventional Hydrogen Bond
	A:PHE289 - N:UNK1	5.01893	Hydrophobic	Pi-Pi T-shaped
	A:PHE377 - N:UNK1	5.43843	Hydrophobic	Pi-Pi T-shaped

	A:PHE377 - N:UNK1:C	4.93806	Hydrophobic	Pi-Alkyl
	A:TRP386 - N:UNK1:C	5.25696	Hydrophobic	Pi-Alkyl
	N:UNK1 - A:ILE166	5.30112	Hydrophobic	Pi-Alkyl
	N:UNK1 - A:ILE285	4.40702	Hydrophobic	Pi-Alkyl
4-Hydroxybenzoic acid (**7**)	A:GLN159:HE21 - N:UNK1:O	2.42414	Hydrogen Bond	Conventional Hydrogen Bond
	N:UNK1:H - A:ASN315:OD1	2.26254	Hydrogen Bond	Conventional Hydrogen Bond
	N:UNK1:H - A:GLU378:OE2	2.12521	Hydrogen Bond	Conventional Hydrogen Bond
	N:UNK1 - A:ILE285	4.03797	Hydrophobic	Pi-Alkyl
4-Hydroxybenzaldehyde (**8**)	A:ASN315:HD21 - N:UNK1:O	2.10624	Hydrogen Bond	Conventional Hydrogen Bond
	A:ASN413:HD21 - N:UNK1:O	2.70796	Hydrogen Bond	Conventional Hydrogen Bond
	A:ASN413:HD22 - N:UNK1:O	2.94407	Hydrogen Bond	Conventional Hydrogen Bond
	N:UNK1:H - A:ASN315:OD1	2.88038	Hydrogen Bond	Conventional Hydrogen Bond
	N:UNK1:H - A:GLU378:OE2	2.02856	Hydrogen Bond	Conventional Hydrogen Bond
	N:UNK1 - A:ILE285	4.09051	Hydrophobic	Pi-Alkyl
Glibenclamide	A:ASN32:HD21 - N:UNK1:O	2.19182	Hydrogen Bond	Conventional Hydrogen Bond
	A:ASN286:HD21 - N:UNK1:O	2.44125	Hydrogen Bond	Conventional Hydrogen Bond
	A:ASN286:CA - N:UNK1:O	3.67123	Hydrogen Bond	Carbon Hydrogen Bond
	N:UNK1:C - A:ASN286:OD1	3.53833	Hydrogen Bond	Carbon Hydrogen Bond
	A:GLY417:C,O;LEU418:N - N:UNK1	4.06449	Hydrophobic	Amide-Pi Stacked
	A:ALA68 - N:UNK1:Cl	3.7508	Hydrophobic	Alkyl
	A:ILE285 - N:UNK1	5.2546	Hydrophobic	Alkyl
	N:UNK1:Cl - A:LEU418	3.9955	Hydrophobic	Alkyl
	A:TYR290 - N:UNK1:C	4.22531	Hydrophobic	Pi-Alkyl
	A:PHE414 - N:UNK1:Cl	4.03962	Hydrophobic	Pi-Alkyl
	N:UNK1 - A:VAL67	5.31698	Hydrophobic	Pi-Alkyl
	N:UNK1 - A:ALA68	4.51755	Hydrophobic	Pi-Alkyl

Table S7: Interactions of amino acids of the kappa opioid receptor (PDB ID: 6VI4) with the isolated compounds (**1** to **8**) from *Wendlandia tinctoria* (Roxb.) DC. and the standard drug loperamide during molecular docking.

Compound	Bond (AA and Ligand)	Bond length	Bond type	Bond nature
Liquiritigenin (**1**)	A:ASN286:HD22 - N:UNK1:O	2.69255	Hydrogen Bond	Conventional Hydrogen Bond
	A:TRP386:HE1 - N:UNK1:O	2.51237	Hydrogen Bond	Conventional Hydrogen Bond
	A:ASN413:HD21 - N:UNK1:O	2.13081	Hydrogen Bond	Conventional Hydrogen Bond
	N:UNK1:H - A:GLY382:O	2.92491	Hydrogen Bond	Conventional Hydrogen Bond
	A:PHE377 - N:UNK1	5.21554	Hydrophobic	Pi-Pi T-shaped
	N:UNK1 - A:ILE166	5.25385	Hydrophobic	Pi-Alkyl
	N:UNK1 - A:ILE285	4.96032	Hydrophobic	Pi-Alkyl
Naringenin (**2**)	N:UNK1:H - B:ILE96:O	2.53534	Hydrogen Bond	Conventional Hydrogen Bond
	B:TRP183 - N:UNK1	3.74398	Hydrophobic	Pi-Pi Stacked
	B:TRP183 - N:UNK1	4.20626	Hydrophobic	Pi-Pi Stacked
	N:UNK1 - B:ILE180	5.4624	Hydrophobic	Pi-Alkyl
Apigenin (**3**)	N:UNK1:H - B:ILE96:O	2.53534	Hydrogen Bond	Conventional Hydrogen Bond
	B:TRP183 - N:UNK1	3.74398	Hydrophobic	Pi-Pi Stacked
	B:TRP183 - N:UNK1	4.20626	Hydrophobic	Pi-Pi Stacked
	N:UNK1 - B:ILE180	5.4624	Hydrophobic	Pi-Alkyl
Kaempferol (**4**)	B:PHE99 - N:UNK1	5.40779	Hydrophobic	Pi-Pi Stacked
	B:TRP183 - N:UNK1	3.74362	Hydrophobic	Pi-Pi Stacked
	B:TRP183 - N:UNK1	3.95697	Hydrophobic	Pi-Pi Stacked
	B:TRP183 - N:UNK1	4.87118	Hydrophobic	Pi-Pi Stacked
	B:TRP183 - N:UNK1	3.90906	Hydrophobic	Pi-Pi Stacked
Glabridin (**5**)	B:TRP183 - N:UNK1	3.84787	Hydrophobic	Pi-Pi Stacked
	B:TRP183 - N:UNK1	3.94883	Hydrophobic	Pi-Pi Stacked
	B:TYR140 - N:UNK1	4.75559	Hydrophobic	Pi-Pi T-shaped
	N:UNK1:C - B:ILE180	3.94788	Hydrophobic	Alkyl
	N:UNK1:C - B:ILE180	4.57153	Hydrophobic	Alkyl
	B:TRP183 - N:UNK1	5.25665	Hydrophobic	Pi-Alkyl
	B:TRP183 - N:UNK1	4.82862	Hydrophobic	Pi-Alkyl
	B:TRP183 - N:UNK1:C	4.58348	Hydrophobic	Pi-Alkyl
	B:TRP183 - N:UNK1	4.33359	Hydrophobic	Pi-Alkyl
Ferulic Acid (**6**)	N:UNK1:C - B:LYS176	3.89225	Hydrophobic	Alkyl
	N:UNK1:C - B:ILE180	4.87741	Hydrophobic	Alkyl
4-Hydroxybenzoic acid (**7**)	N:UNK1:H - B:ASN179:O	2.26424	Hydrogen Bond	Conventional Hydrogen Bond
	N:UNK1:H - B:ILE96:O	2.0668	Hydrogen Bond	Conventional Hydrogen Bond
	B:TRP183:CD1 - N:UNK1:O	3.3447	Hydrogen Bond	Carbon Hydrogen Bond
4-Hydroxybenzaldehyde (**8**)	B:ILE180:CA - N:UNK1:O	3.42981	Hydrogen Bond	Carbon Hydrogen Bond
	B:TRP183:CD1 - N:UNK1:O	3.57398	Hydrogen Bond	Carbon Hydrogen Bond
Loperamide	N:UNK1:C - B:TRP183	3.8229	Hydrophobic	Pi-Sigma

	B:TRP183 - N:UNK1	5.13627	Hydrophobic	Pi-Pi Stacked
	N:UNK1:Cl - B:ILE96	4.88724	Hydrophobic	Alkyl
	B:PHE99 - N:UNK1:Cl	5.38651	Hydrophobic	Pi-Alkyl
	B:TRP183 - N:UNK1	5.04811	Hydrophobic	Pi-Alkyl
	N:UNK1 - B:LEU107	5.2433	Hydrophobic	Pi-Alkyl
